# Supplementary material for: Preparation and structure of SiOCN fibres derived from cyclic silazane/poly-acrylic acid hybrid precursor
Source: R Soc Open Sci. 2019 Oct 2;6(10):190690. doi: 10.1098/rsos.190690 (PMC6837217; doi:10.1098/rsos.190690)
Supplement: Supplementary Figures [file rsos190690supp1.docx]

**Preparation and Structure of SiOCN Fiber Derived from Cyclic Silazane/PAA Hybrid Precursor**

Zhongkan Ren, Christel Gervais, Gurpreet Singh

**Supplemental Material**


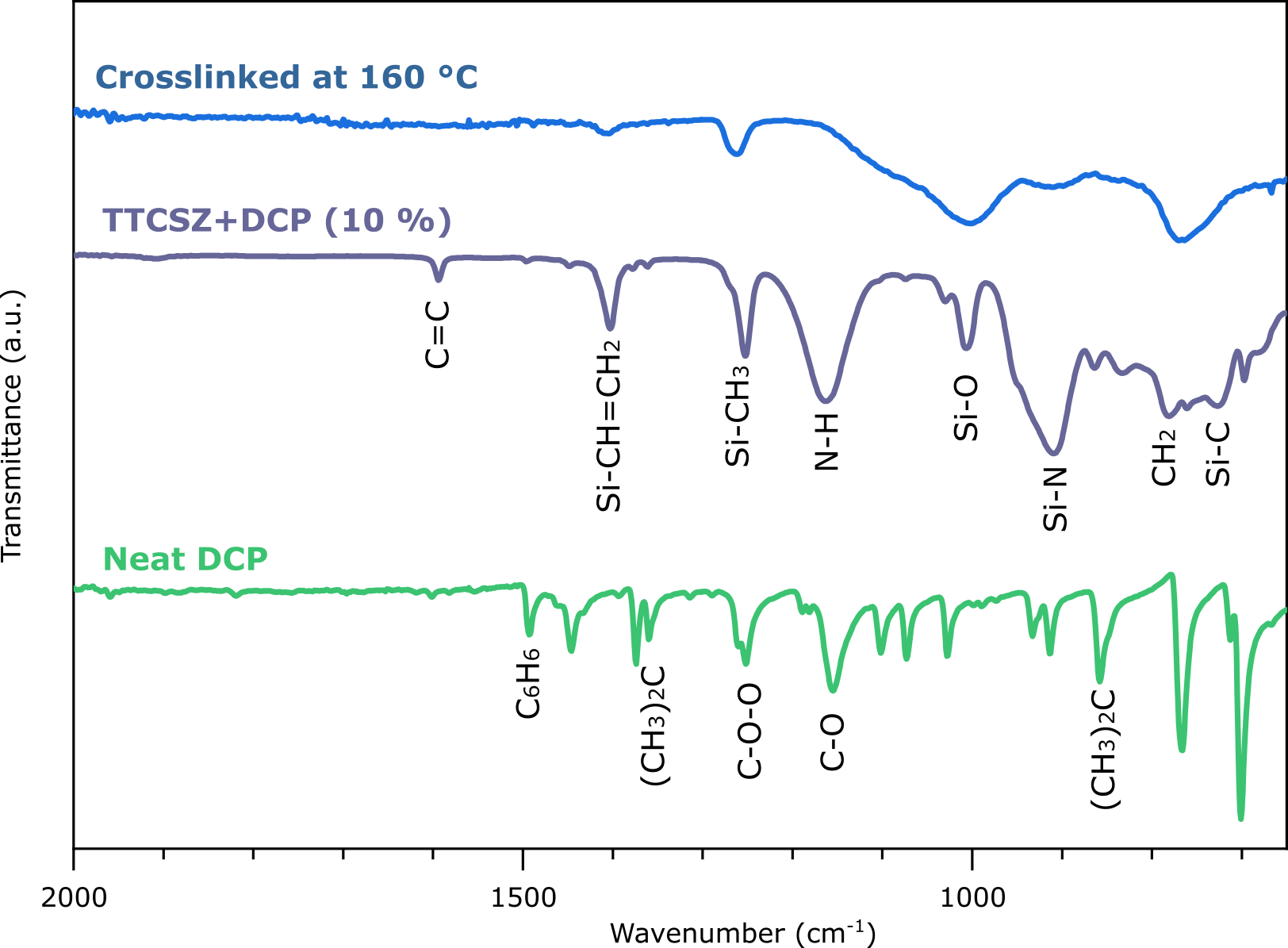


**Figure S1. Influence of DCP on FTIR signal.** The FTIR spectra of neat DCP, TTCSZ+DCP (10 wt. %) and TTCSZ+DCP (10 wt. %) crosslinked at 160 °C are presented.

In order to investigate the effect of DCP on FTIR signals, we performed additional FTIR analysis on neat dicumyl peroxide (DCP), TTCSZ + DCP (10 wt. %) mixture and crosslinked TTCSZ + DCP (10 %). DCP is not detectable from the FTIR of TTCSZ+DCP mixture (the signature peaks are extremely weak). Especially after crosslinking at 160 °C, DCP signal became completely undetectable by FTIR. Hence, it is fair to assume that the DCP is washed away or decomposed at the crosslinking stage.


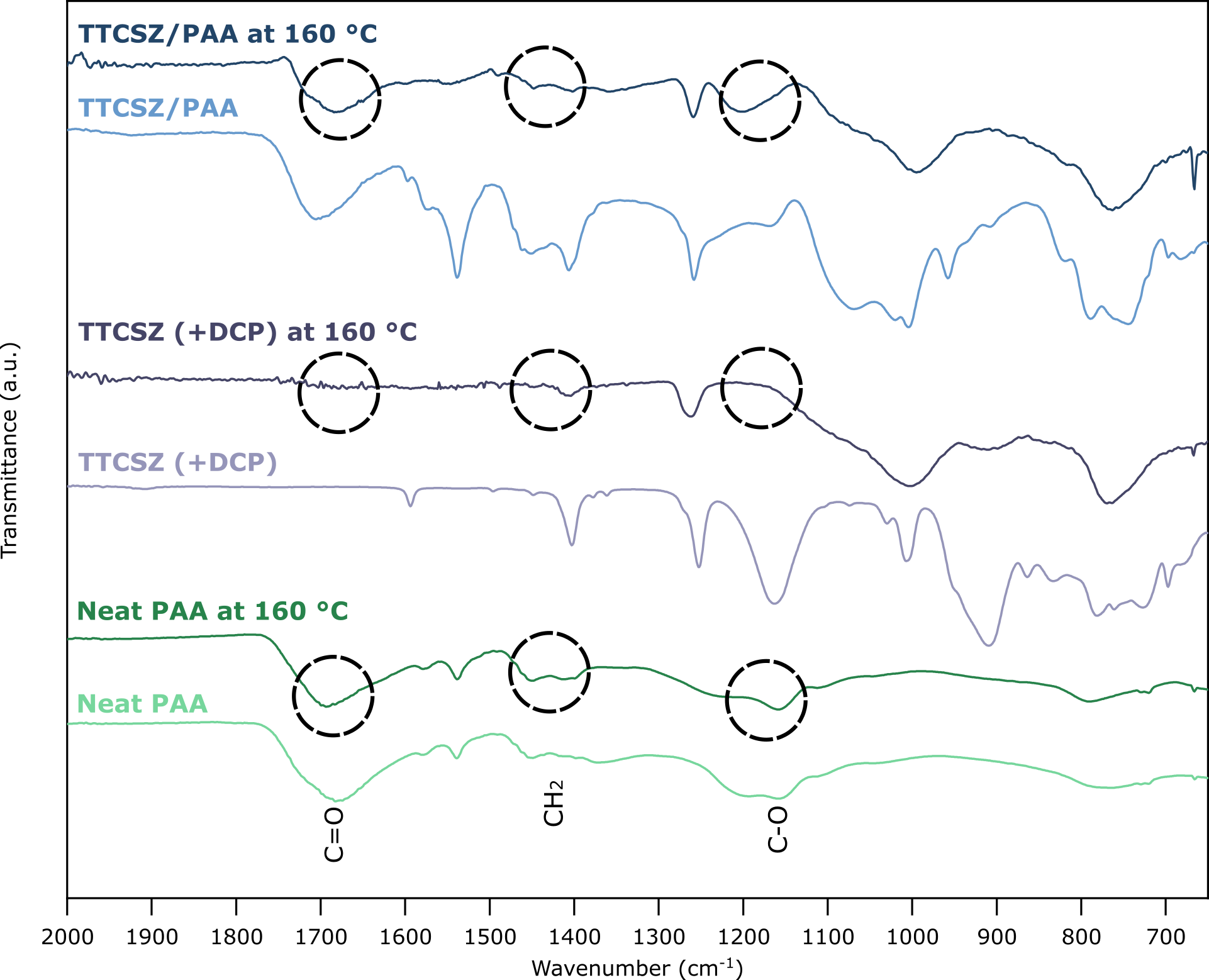


**Figure S2. Weak or no crosslinking between PAA and TTCSZ.** FTIR spectra of neat PAA, TTCSZ (DCP 10 wt. %) and TTCSZ/PAA and corresponding heat treated samples.

We performed FTIR on TTCSZ (10 wt% DCP) and crosslinked TTCSZ (10 wt% DCP)---Fig. S2. When comparing this result with previous FTIR data, several points can be made:

(1) Neat PAA and PAA heated at 160 °C did not show significant changes in FTIR spectrum.

(2) Comparing TTCSZ/PAA crosslinked at 160 °C and TTCSZ crosslinked at 160 °C without the PAA, the main differences lie at the PAA peaks (C=O, CH_2_ and C-O peak, circled in the figure).

Based on the abovementioned points, introduction of PAA in to TTCSZ had little or no effect on the chemical structure of TTCSZ. Hence, it is fair to conclude that no chemical reaction took place between PAA and TTCSZ during the physical mixing at room temperature or during heating at160 °C.


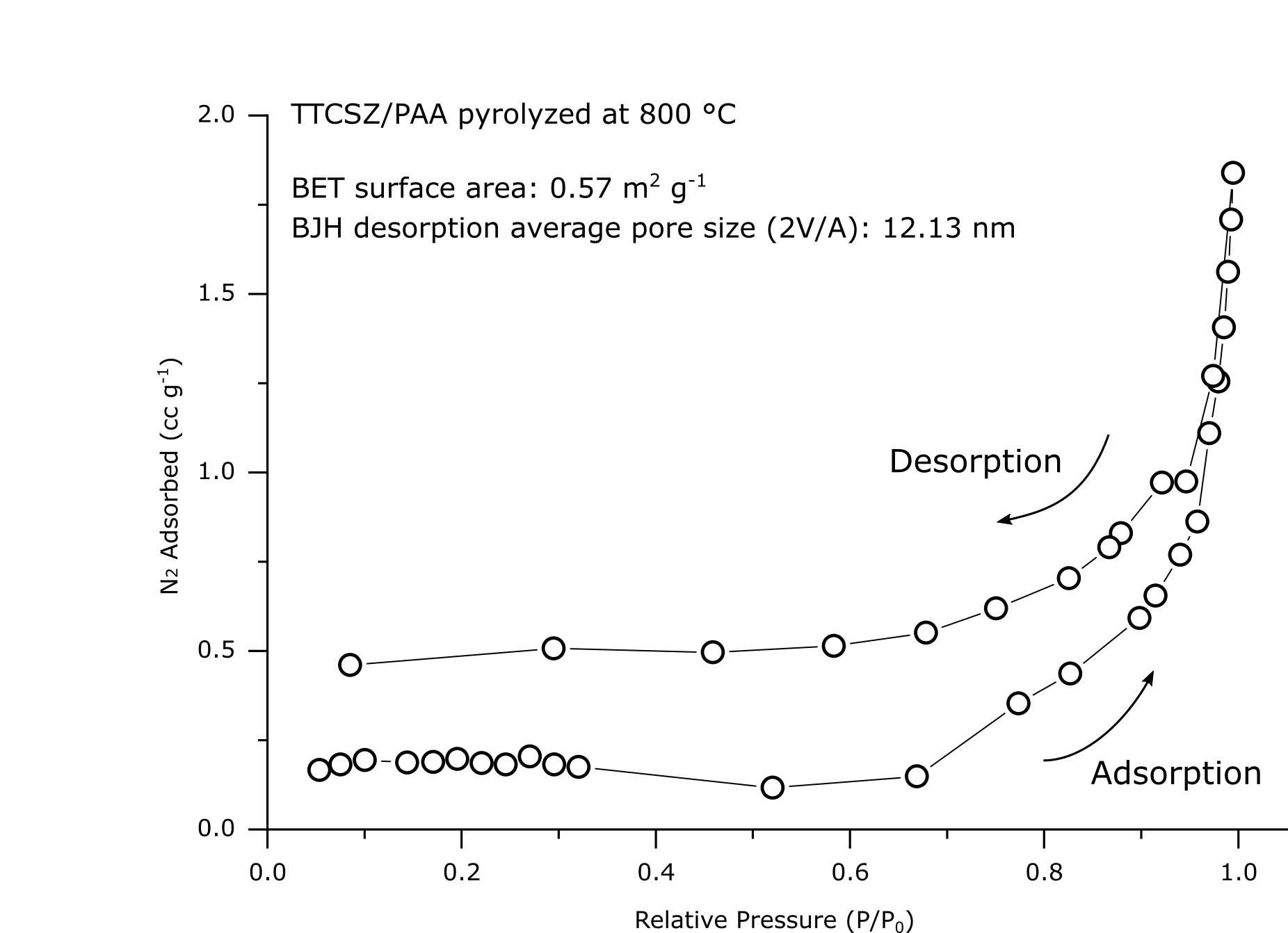


**Figure S3. BET analysis result on TTCSZ/PAA pyrolyzed at 800 °C.** The data plotted shows the N_2_ adsorption/desorption isotherm of the fiber samples with BET surface area and average pore size shown.

Additional BET analysis was performed on the pyrolyzed fiber samples using ASAP 2460 Surface Area and Porosimetry Analyzer (Micromeritics) in N_2_ at 77.3 K. The corresponding results are shown in the Fig S3. The BET analysis results show a moderate BET surface area of 0.57 m^2^ g^-1^.
